# Body representation difficulties in children and adolescents with autism may be due to delayed development of visuo-tactile temporal binding

**DOI:** 10.1016/j.dcn.2017.04.007

**Published:** 2017-04-27

**Authors:** Danielle Ropar, Katie Greenfield, Alastair D. Smith, Mark Carey, Roger Newport

**Affiliations:** a School of Psychology, University of Nottingham, University Park, Nottingham NG7 2RD, UK; b Department of Psychology, University of York, Heslington, York YO10 5DD, UK

**Keywords:** Autism spectrum disorder, Temporal binding window, Visuo-tactile processing, Embodied action

## Abstract

Recent research suggests visuo-tactile binding is temporally extended in autism spectrum disorders (ASD), although it is not clear whether this specifically underlies altered body representation in this population. In the current study children and adolescents with ASD, and typically developing controls, placed their hand into mediated reality system (MIRAGE) and saw two identical live video images of their own right hand. One image was in the proprioceptively correct location (veridical hand) and the other was displaced to either side. While visuo-tactile feedback was applied via brushstroke to the participant’s (unseen) right finger, they viewed one hand image receiving synchronous brushstrokes and the other receiving brushstrokes with a temporal delay (60, 180 and 300 ms). After brushing, both images disappeared from view and participants pointed to a target, with direction of movement indicating which hand was embodied. ASD participants, like younger mental aged-matched controls, showed reduced embodiment of the spatially incongruent, but temporally congruent, hand compared to chronologically age-matched controls at shorter temporal delays. This suggests development of visuo-tactile integration may be delayed in ASD. Findings are discussed in relation to atypical body representation in ASD and how this may contribute to social and sensory difficulties within this population.

Although Autism Spectrum Disorders (ASD) have primarily been characterised by difficulties with social communication, interaction, and imagination (Wing and Gould, 1979), atypical sensory processing has recently become a greater focus for identifying and understanding individuals with autism (DSM-V; American Psychological Association, 2013). Clinical reports (e.g. Leekam et al., 2007, Talay-Ongan and Wood, 2000) have documented sensory abnormalities in over 90% of individuals with ASD, highlighting its significance as a defining feature in this population.

Despite the prevalence of atypical sensory processing in autism, many prominent theories of ASD, such as Theory of Mind (Baron-Cohen et al., 1985) and Social Motivation Theory (Chevallier et al., 2012), have focused soley on social interaction difficulties in ASD. Though Weak Central Coherence theory (Happe and Frith, 2006) and Enhanced Perceptual Functioning (Mottron et al., 2006) present a partial explanation for sensory sensitivities, neither theory fully specifies the mechanisms underlying these atypicalities. Furthermore, these theories are unable to account for the heterogeneity of sensory sensitivities seen within and between individuals with ASD, nor can they explain why an individual can exhibit both hyper- and hypo-sensitivities to sensory stimuli (Leekam et al., 2007, Pellicano and Burr, 2012).

Alternatively, it has been suggested that both sensory and socio-communicative features of ASD could be due, at least in part, to atypical multisensory integration (MSI) (Brock et al., 2002, Cascio et al., 2012, Stevenson et al., 2014, Foss-Feig et al., 2010, Kwakye et al., 2011). Evidence from the typical population suggests that MSI develops over a protracted period of time throughout early childhood and becomes more sensitive and specific with age (Gori et al., 2008, Nardini et al., 2008, Cowie et al., 2013, Cowie et al., 2016). As the social world requires one to efficiently integrate sensory information from a range of sources (e.g. auditory, visual, tactile, proprioception), difficulties in binding related inputs could lead to impaired social interaction and sensory overload. For instance, communicating with another person necessitates detecting the temporal synchrony between their speech and lip movements. At the same time one also needs to be able to exclude extraneous sensory information that is unrelated to the event (e.g. the sound of a television in the background). If temporal binding is extended or less precise in ASD then this would lead to problems distinguishing the synchronous sensory information relating to the speaker from sensory inputs that originated from unrelated stimuli (Bahrick and Todd, 2012). In support of this argument, Stevenson et al. (2014) demonstrated a relationship between temporally extended audio-visual binding and poor speech processing abilities in children with ASD. Whilst this research explains how communication difficulties in ASD could result from atypical audio-visual binding, there has been a limited amount of research exploring the temporal processing of other sensory modalities in ASD.

One area of sensory integration that merits further research is visuo-tactile-proprioceptive processing. Accurate integration of visual, tactile and proprioceptive inputs underlies our sense of bodily self (i.e. body representation), including body localisation (the ability to locate our limbs) and a sense of body ownership (the awareness and understanding that our body belongs solely to us, and that we can see, feel and move it) (Gallagher, 2000, Nava et al., 2014). Body localisation and body ownership are both important for identifying, distinguishing and comparing ourselves with others (Meltzoff, 2007, Schutz-Bosbach et al., 2006). For instance, many researchers have argued that the ability to detect similarities between someone else’s movements and our own is a foundation for perspective taking and empathy for others as it involves ‘mentally standing in their shoes’ (Husserl, 2012, Smith, 2010). Thus, if visuo-tactile-proprioceptive integration is not developing typically, then this could affect the development of one’s bodily self, impacting on various higher-order social processes. In support of this, a recent study (Pearson et al., 2016) exploring mechanisms underlying visual perspective taking found performance in typically developing children was predicted by good performance on a body representation task, however this was not the case for those with ASD. Furthermore, there has been evidence of atypical body representation being related to poor empathy in children with autism (Cascio et al., 2012).

Although there appears to be a clear case for the importance of body representation in social processes, only recently has research demonstrated that extended temporal binding of visuo-tactile inputs may underlie atypical development of the bodily self (Greenfield et al., 2015). Greenfield et al. (2015) developed a task which manipulated visuo-tactile and spatial input in order to induce ownership of a virtual hand. Children and adolescents with ASD and typically developing controls placed their right hand into a multisensory illusion apparatus (MIRAGE, University of Nottingham), which presented two identical live video images of their own hand, immediately above the location of the actual hand and in the same plane as the actual hand. One virtual hand was always aligned proprioceptively with the actual hand (called the veridical hand) and the other was displaced to the left or right of this. While a brush stroke was applied to the participant’s actual (hidden) hand, they observed the two virtual images of their hand also being stroked, only one of which had synchronous visuo-tactile inputs while for the other the seen and felt brush strokes were temporally asynchronous. Participants were asked to identify which seen hand was their actual hand subjectively. One approach to performing the task would be to ignore the visuo-tactile input provided by the brush stroking and rely solely on proprioceptive information. However, a wealth of evidence has demonstrated that visuo-tactile synchrony can override proprioceptive information and induce the sense of ownership over a fake limb (see Makin et al., 2008, Tsakiris, 2010). Therefore, detection of temporal synchrony between the felt brush stroke on the participant’s actual (unseen) hand and seen brush stroke on either of the virtual hands is essential to body ownership. In order to test for sensitivity to temporal information between visual-tactile inputs, Greenfield et al. (2015) administered a delay of either 60 ms, 180 ms, or 300 ms. Typical, chronologically-matched participants were more consistent than those with ASD i in reporting the synchronous hand to be their real hand at shorter delay lengths (60 ms), even when the image of the synchronous hand was visually displaced from the location of the real hand. These results were interpreted as showing that visual-tactile binding occurs over an extended period of time in autistic children which suggests that the typical integration processes underlying body representation are disrupted. These findings are consistent with other research with individuals with ASD showing reduced susceptibility to the rubber hand illusion which also requires visual-tactile integration (Cascio et al., 2012, Paton et al., 2012).

Whilst the study by Greenfield et al. (2015) demonstrated that participants with ASD had greater difficulties in associating visual-tactile synchrony with their own body at shorter delays, the findings are perhaps limited by the fact that they were based on subjective, forced-choice reports of ownership which only give a categorical measure and cannot tell us the extent to which temporal synchrony affects body ownership in ASD. Furthermore, as individuals with ASD can be overliteral in their interpretation of language (Happe, 1995) it is possible that this could have at least partly contributed to the findings. For instance, when asked “which hand is your actual hand” when viewing the two identical virtual hand images an overliteral interpretation could have resulted in one thinking neither were or both were their real hand.

In addition, the subjective feeling of ownership may not accurately reflect whether the ‘owned’ body part is incorporated into the body schema (an unconscious representation of the body that is used for action and interaction with the environment) rather than body image (a top-down, perceptual representation of the body) (Haggard and Wolpert, 2005, Kammers et al., 2010, Kammers et al., 2006, Kammers et al., 2009). In an almost identical task in healthy adults, Newport et al. (2010) demonstrated that the hand stroked in visual-tactile synchrony is incorporated into both body image and body schema. Evidence that body image and schema can be dissociated in this task, however, was later demonstrated in a patient with visuo-spatial neglect who consistently chose different fake hands for subjective ownership (body image) and target pointing (body schema) (Preston and Newport, 2011).

In terms of understanding our own body and actions, in order to understand those of others, an investigation of body schema may be more important and more revealing than body image given the evidence that we understand others’ actions through the actions of the self (Chaminade et al., 2005, Gallese, 2003, Gallese et al., 2004). Thus, it might be reasonable to assume that an inability to effectively use temporally synchronous sensory information to construct their own body schema for those with ASD would have a knock-on effect for their ability to understand the social body cues of others. For that reason, the current study retested the same population as in Greenfield et al., 2015, but on a task that directly measured the effect of temporal binding on the body schema. For this task, after seeing two images of their right hand being stroked (one synchronous and one with delay), participants were required to point to a target with their real, unseen hand. The degree to which the synchronously stroked hand had been incorporated into body schema can be inferred from the direction and magnitude of pointing errors. If participants with ASD do not integrate visual and tactile sensory input across the same temporal delays as typically developing individuals then this will result in a pointing trajectory that reflects embodiment of the spatially congruent hand across all conditions. In typically developing children and adolescents it is expected that temporal synchrony will provide the basis for updating the body schema and will be tightly bound to the image of the hand with visual-tactile synchrony, even when their actual hand is in a different spatial location. Therefore, control participants should show pointing trajectories indicating they have incorporated the virtual hand with synchronous visuo-tactile input regardless of its spatial congruency.

## 1 Method

### 1.1 Participants

All participants in this study had also taken part in a previous published study carried out by the same authors (Greenfield et al., 2015). Participants included 31 children and adolescents with ASD, aged 8–15 years (two female, one left-handed), 28 chronological age-matched (CA) typically developing controls (8 female, 5 left-handed), and 27 verbal mental age-matched (MA) typically developing controls, aged 5–10 years (10 female, 2 left-handed). Individuals with ASD were recruited from autism support groups and a specialist autism unit within a local school in Nottingham. Comparison participants were recruited from Summer Scientist Week (n = 40), a community event held at the University of Nottingham, or from the University’s database of local families (n = 18). As evidence has shown temporal binding processes are refined and become more sensitive with age, (Hillock-Dunn and Wallace, 2012) the ASD group was matched to both a group of chronologically age-matched and a group of verbal-mental age-matched controls. The British Picture Vocabulary Scale-III (BPVS-III; Dunn and Dunn, 2009) was administered to assess level of receptive understanding of language so that those with ASD could be matched with a verbal mental age control group. There were no significant differences in verbal mental age between the ASD and MA group, or in chronological age between the ASD and CA group. The individuals with ASD varied in their cognitive abilities and we therefore calculated developmental quotient (DQ) scores (Chaoying et al., 1999) to give an indication of the range of delay in the group (see Table 1). The parents of all participants gave written informed consent prior to testing and ethical approval for the experiment was granted by the University of Nottingham, School of Psychology Research Ethics Committee and was conducted in accordance with the ethical standards of the Declaration of Helsinki.

**Table 1:** Participant descriptives for chronological age (CA) matched, verbal mental age (MA) matched and autism spectrum disorder (ASD) groups. Abbreviations: SAS- Social Aptitudes Scale; SCQ- Social Communication Questionnaire; DQ- Developmental Quotient.

Group (sample size)	Statistic	Age in months	Verbal mental age in months	SAS	SCQ	DQ
ASD (29)	Mean	151.65	103.17	10	24.64	69
	SD	23.07	37.37	5.90	5.2	24.43
	Min	99.72	59.00	0	15	38.10
	Max	191.04	189.00	23	34	134.04

MA matched (27)	Mean	95.29	101.56	26.13	Not collected	N/A
	SD	16.99	27.86	7.73		
	Min	64.00	64.00	19		
	Max	123.6	172.00	39		

CA matched (28)	Mean	152.18	147.69	24.71	Not collected	N/A
	SD	19.85	32.8	6.17		
	Min	116.76	101.00	13		
	Max	184	189.00	40		

All individuals in the ASD group had received a previous diagnosis of autism, autism spectrum disorder, or Asperger Syndrome, by an independent clinician employed by the National Health Service using the Autism Diagnostic Observation Scale (ADOS; Rutter et al., 2012) or the Autism Diagnostic Interview (ADI-R; Rutter et al., 2005). Confirmation of diagnosis was obtained by the researchers via a parent/caregiver in a background questionnaire and additionally through parents’ ratings on the Social Communication Questionnaire (SCQ; Rutter and Lord, 2003) and the Social Aptitude Scale (SAS; Liddle et al., 2008). Parents of two individuals did not return the completed questionnaires; however, since participants in the ASD group were recruited from a specialist Autism unit requiring a formal diagnosis and statement of special educational needs, it is very unlikely they did not have ASD. Individuals in all groups were screened for other developmental difficulties (e.g. motor, attention, visual, language delay) via a parental background questionnaire. None of the typically developing participants had a diagnosis of ASD or any other learning difficulty, confirmed by parent questionnaire and additional screening measures. In the ASD group one individual had dyspraxia, one had dyslexia, one had ADHD, and one was reported to have hypermobile joints.

There were several criteria participants were required to meet to be included in the study. Firstly, all needed to have normal or corrected-to-normal vision. Secondly, all participants took part in practice trials in which they needed to demonstrate: (1) an ability to keep their hand still and (2) comprehension of the task. Two individuals from the ASD group were excluded, as they could not keep their hand still to complete the task, leaving 29 participants with ASD whose results were included in the analyses (see Table 1 for participant descriptives).

### 1.2 Procedure

**Fig. 1:**
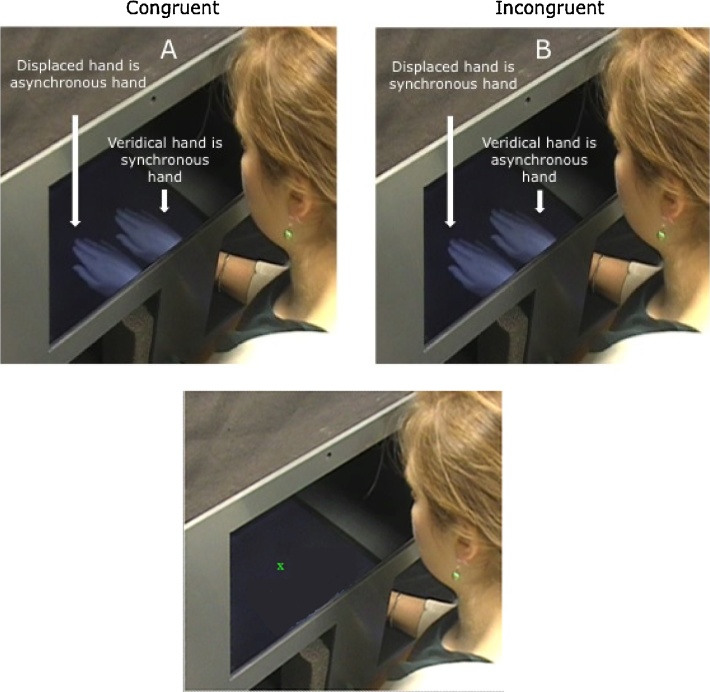
Mirage Task. Participants placed their right hand into the MIRAGE and saw two live video images of the hand. The veridical hand was in the same location as the actual hand; the displaced hand was to the left or right of the veridical hand. In (A) and (B) the arm is in view for illustrative purposes, but was covered in the experiment so participants could not see the relationship between their limb and the images. (A) In spatially congruent conditions, the displaced hand had a temporal delay of with 60, 180, 0r 300 ms applied to it; the veridical hand did not (synchronous hand). (B) In spatially incongruent conditions the veridical hand had a temporal delay of either 60, 180. Or 300 ms applied to it; the displaced hand did not (synchronous hand). (C) After 10 s of brushing, the screen went blank and participants pointed with their real hand at a target (green cross) located between the two previously presented hand images. (For interpretation of the references to color in this figure legend, the reader is referred to the web version of this article.)

Participants were tested in a quiet room at the University or their school. All completed a body ownership task, conducted using the MIRAGE device (Newport et al., 2010). This task took approximately 15 min, and was either preceded or followed by the BPVS. Breaks were provided if needed. MIRAGE presents live video images of the hand in real time as if viewing the hand directly; that is, in the same spatial location and from the same visual perspective. Depending on their height, participants sat or knelt on a chair to allow them to comfortably view their right hand when they placed it onto the work surface of the MIRAGE. A rectangular black bib was attached across the length of the MIRAGE, on the side that the participant was seated, to obscure the work surface from view. Participants wore a black adjustable sleeve, which covered their right wrist and forearm, ensuring that only the hand was visible when their arm was in the MIRAGE. Participants placed their right hand into the device and saw two virtual representations of their hand: the veridical hand was in the same location as the participant’s actual hand while the displaced hand was immediately to the left or right of this (see Fig. 1). Participants first completed practice trials, which were identical to experimental trials described below except that neither hand image showed a visual-tactile delay. These were included to ensure that participants were comfortable with the set-up and understood the task requirements.

In the experimental trials, the participant’s right index finger was brushed at 1 Hz for 10 s while they observed the brushstrokes on both virtual right hand images. In spatially congruent conditions the veridical hand was stroked synchronously, while the displaced hand had a temporal delay of either 60, 180 or 300 ms applied to it. In spatially incongruent conditions the displaced hand was stroked synchronously, whereas the veridical hand had a temporal delay of either 60, 180 or 300 ms applied to it. After brushing, both hand images disappeared from view and a target (a green cross) was presented on the screen for five seconds. This appeared half way between the two previously-presented hand images, aligned horizontally with the tip of the index fingers (see Fig. 1). For each condition, the displaced hand was presented once to the left of the veridical hand and once to the right of it (counterbalanced across conditions). The target was thus presented to the left of the participants’ actual index finger in half the conditions and to the right in the remaining conditions. Participants were asked to point at the green cross, quickly and accurately, with their real right index finger and to hold this position until the target disappeared (5-s duration). The MIRAGE device recorded participants’ hand movements during this phase, allowing for later calculation of pointing accuracy (with fidelity at the level of individual pixels). Vision of the hand remained occluded whilst the experimenter placed the participant’s hand at the starting point for the next trial. The start point for each trial was identified by a red cross superimposed on the image of the MIRAGE workspace that was visible to the experimenter on their computer, but not visible to the participant. In total, there were two trials for each of the six conditions: spatially congruent 60 ms, 180 ms and 300 ms delay; and, spatially incongruent 60 ms, 180 ms and 300 ms delay. Trial order was fully randomised for each participant. While we acknowledge two trials are not ideal for response reliability, it was more important, given the characteristics of the participants, to keep the experiment brief to ensure attention was maintained so that responses accurately reflected performance on the task.

## 2 Results

### 2.1 Data analysis

**Fig. 2:**
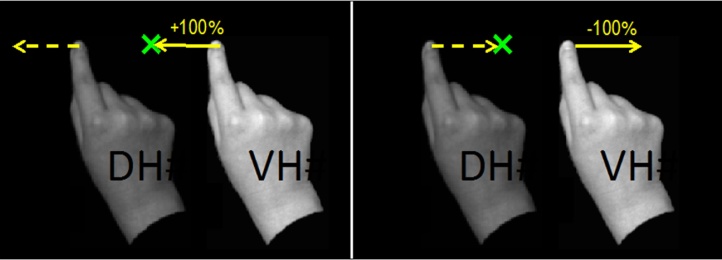
Left Panel, embodiment of the veridical hand: A score of 100 (solid arrow) equates to pointing exactly to the target with a trajectory as though the real hand were in the location of the veridical hand (VH). Scores above 100 indicate over-reaches i.e. pointing in the direction of the target but beyond it. Right Panel, embodiment of the duplicate hand: pointing as though the real hand were in the location of the displaced hand (DH) (dashed arrow) would result in the real hand (which is actually in the same location as the VH) moving away from the target (solid arrow) and being given a negative score. Note that neither hand was visible at the time of reaching and that the displaced hand (DH) is depicted as less vivid than the Veridical Hand for pictorial purposes only.

Participants’ hand movements were recorded during the five-second duration that the target appeared on the screen. For each video clip, the x-axis coordinates of three locations were recorded in pixels (1 pixel = 0.75 mm): (1) the tip of the index finger at the start of the video (baseline measurement), (2) the tip of the index finger at the end of the video (pointing measurement) and (3) the centre of the target. These values were entered into a Labview programme to calculate the distance and direction of reaches for each trial. For each condition, the target appeared once to the left of the veridical hand and once to the right of it. Embodiment of the veridical hand would lead to a pointing response with the real hand in the direction of the target, whereas embodiment of the displaced hand would lead to a pointing response in the opposite direction, away from the target. To facilitate analysis, errors were calculated as negative if participants pointed away from the target with their real hand, regardless of whether the target was to the left or right of the veridical hand. A score of 100 equates to pointing exactly on the target with the veridical hand, a score of −100 would indicate full embodiment of the displaced hand in the spatially incongruent condition (Fig. 2).

2.6% of the total dataset was missing due to a technical error when recording the videos. Missing data was dealt with using casewise deletion leaving 25 ASD, 26 CA-matched and 22 MA-matched participants whose data was included in the analysis. For the remaining participants, the CA and ASD groups were not significantly different on CA (p = 0.619) and the MA and ASD groups were not significantly different on MA (p = 0.944).

Bonferroni corrected (p < 0.003) one-sampled *t*-tests against 100 (equating to pointing directly on the target) were conducted for each group, at each condition to give an indication of accuracy. To assess the extent to which asynchronous visuo-tactile inputs affected embodiment, scores in spatially congruent conditions were subtracted from scores in incongruent conditions for each group at each delay length. Thus, a congruency score of 0 would equate to their being no switch from using the spatially congruent hand to the incongruent hand (that is, no effect of synchronicity on hand embodiment). Positive scores represent a switch or relocation in the direction of the synchronous hand and negative scores a switch to the asynchronous hand. One would expect a high positive score if hand embodiment were driven by the detection of temporal multisensory congruence. These congruency scores were entered in a repeated measures ANOVA with group (CA versus MA versus ASD) as the between-subjects factor and delay (60 ms versus 180 ms versus 300 ms) as the within-subjects factor. Assumptions for normality, homogeneity and sphericity were all met unless otherwise stated. All analyses were re-run without outliers as determined by the outlier labelling rule using 2.2 as a multiplier (Hoaglin and Iglewicz, 1987). The pattern of results remained the same, and the results reported below therefore include outliers.

### 2.2 Data

**Fig. 3:**
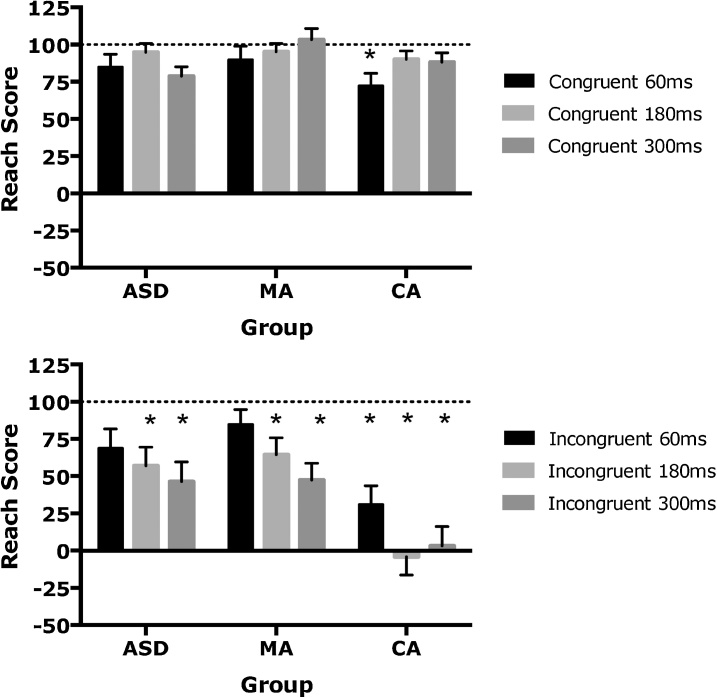
Mean reach scores for autism spectrum disorder (ASD), verbal mental age (MA) matched and chronological age (CA) matched control groups. Error bars show standard error of the mean. A score of 100 equates to pointing directly on the target with the veridical hand (dotted line). *Indicates scores that are significantly different from 100 at p < 0.003.

Mean reach scores for each group in each condition are displayed in Fig. 3. In the spatially congruent condition, pointing accuracy was very good across groups showing scores close to the actual target location (i.e. 100), with the exception of the CA group in the 60 ms delay condition. One-sampled *t*-tests (Bonferroni-corrected) confirmed that scores were only significantly lower than 100 (signifying reduced accuracy) for the CA group in the spatially congruent 60 ms, t(27) = 3.90, p = 0.001). In contrast, performance in the spatially incongruent condition led to a decrease in pointing accuracy with a few exceptions. The CA group showed significantly reduced accuracy across all three delays: 60 ms, t(26) = 5.36, p < 0.001; 180 ms, t(27) = 7.92, p < 0.001; 300 ms conditions, t(26) = 7.65, p < 0.001. For the MA and CA group, scores were significantly lower than 100 only in the spatially incongruent 180 ms condition [MA: t(26) = 4.08, p < 0.001, ASD: t(25) = 3.57, p = 0.001] and 300 ms condition [MA: t(26) = 7.31, p < 0.001: ASD: t(27) = 4.18, p < 0.001]. No other results were significant.

**Fig. 4:**
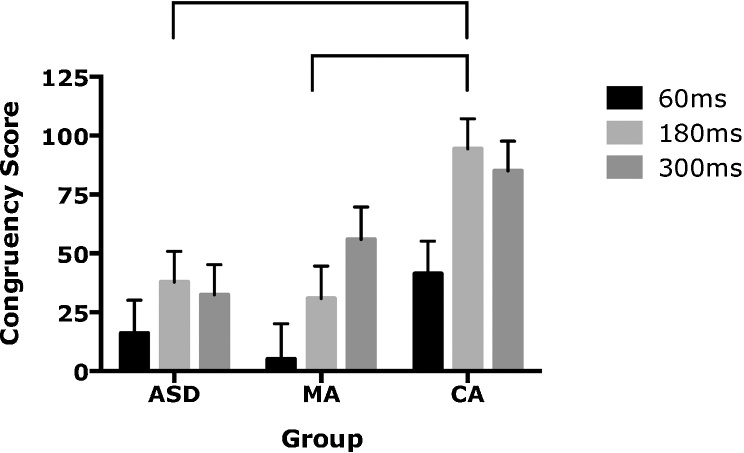
Congruency scores for the autism spectrum disorder (ASD), verbal mental age (MA) matched and chronological age (CA) matched control groups. Error bars represent standard error. Braces indicate Bonferroni-corrected significant group differences.

In order to allow us to compare across groups more easily, a spatial congruency effect was calculated which gives an indication of the extent to which embodiment of the synchronous hand occurred across conditions. The effect of spatial congruency (i.e. incongruent score – congruent) scores is shown in Fig. 4. A score of zero indicates similar performance on the spatially congruent and incongruent conditions (i.e. no embodiment). As performance was generally accurate in the spatially congruent condition for all groups (Fig. 3), higher congruency scores in Fig. 4 represent the extent to which the displaced (synchronous) hand was embodied. The repeated-measures ANOVA found a main effect of delay, F(1.83, 140) = 13.71, p < 0.001. The assumption of sphericity was violated for this effect, as specified by Mauchly’s test, X^2^(2) = 0.91, p = 0.034, and degrees of freedom are therefore reported using Greenhouse-Geisser estimates of sphericity. Pairwise comparisons (Bonferroni corrected) revealed no significant difference between the 180 ms and 300 ms delays (p = 1) but scores were significantly lower at 60 ms compared to 180 ms (p = 0.001) and 300 ms delays (p < 0.001). A main effect of group was also found, F(1,70) = 5.47, p = 0.006. Levene’s test showed that the variance in congruency scores at the 180 ms delay was smaller in the ASD and MA groups compared to the CA group (p = 0.016; see Fig. 4). However, with large sample sizes, Levene’s test can be significant when group variances are not exceptionally different, so corrections were not made for this. Pairwise comparisons (Bonferroni corrected) revealed no significant difference between the ASD and MA groups (p = 1) but spatial congruency scores were significantly higher for the CA group compared to the MA group (p = 0.024) and the ASD group (p = 0.013). No other main effects or interactions were significant.

To explore the relationship between performance on the body representation task, which relies on sensory integration, and social functioning, correlational analyses were carried out. An average spatial congruency score was calculated by averaging across all three temporal delays and correlated with scores on the Social Aptitude Scale across all participants. A small, but significant, positive correlation was found between average congruency scores and performance on the Social Aptitude Scale [r = 0.264, n = 73, p = 0.012 (one-tailed)]. This indicates that those who were given a higher rating on the Social Aptitude Scale, representing better social skills, showed a greater embodiment of the spatially incongruent hand. As Social Communication Scores were only obtained for participants with ASD, a correlation between SCQ scores and average congruency scores was carried out with this group alone. Correlational analyses revealed no significant relationship between these two variables [r = 0.263, n = 22, p = 0.119 (one-tailed)]. In order to explore whether the findings were influenced by some individuals having a cognitive delay, developmental quotient scores were correlated with average congruency performance, however this was not found to be significant (r = 0.067, n = 72, p = 0.287). This suggests that a reduced effect of embodiment cannot be attributed to having a general cognitive delay.

## 3 Discussion

The current experiment assessed whether visuo-tactile integration underlying body representation is temporally extended in children with ASD. Participants pointed to a target following exposure to spatially congruent or incongruent proprioceptive and visuo-tactile inputs for hand ownership. The influence of visuo-tactile cues on body schema (i.e. pointing to a target) was reduced in children with ASD compared to age-matched controls, indicating atypical multisensory abilities relative to their peers. Similar performance between the ASD group with younger but verbal age-matched controls suggests developmental or typical sensory integration processes may be delayed rather than deficit. The specific pattern of results showing the ASD (and MA) participants had particular difficulty in embodying the synchronous hand at the shortest delay is consistent with less precise visuo-tactile temporal binding in these populations. This corresponds with findings from Greenfield et al. (2015) and research in the audio-visual domain suggesting an enlarged temporal binding window (TBW) for sensory integration in children with ASD (Stevenson et al., 2014, Foss-Feig et al., 2010, Kwakye et al., 2011). The finding of the younger typically developing MA group (CA range 5–10) showing less embodiment at shorter temporal delays than the older typically developing group (CA range 10–15), is consistent with other evidence indicating multisensory integration develops across early childhood (Cowie et al., 2013, Cowie et al., 2016, Greenfield et al., 2015, Gori et al., 2008). Explanations for these main findings will be explored below.

In spatially congruent conditions, children in all groups consistently showed pointing movements in the direction of the target indicating they had embodied the veridical hand image that received synchronous visuo-tactile information. Performance in these conditions is in line with typically developing adults (Newport and Preston, 2011) and indicates that the participants understood the task and were able to accurately perform it. However, it was unexpected that accuracy was lower in the spatially congruent 60 ms condition for the CA group compared to the MA and ASD groups (see Fig. 3). Evidence suggests that young typically developing children may show a preference for using unimodal over multimodal information (Gori et al., 2008) which may have put them at an advantage in this condition where the delay was difficult to detect, whereas the older CA group could have been attempting to engage in multisensory processing. This is consistent with research showing that throughout childhood, the ability to integrate multiple sensory inputs develops through experience, leading gradually to optimal MSI by late childhood (Cowie et al., 2013, Cowie et al., 2016, Greenfield et al., 2015, Gori et al., 2008). In fact, it has been shown that by age eleven, at least in relation to processing various depth cues, children show evidence of mandatory fusion (Bedford et al., 2016), suggesting they may not be able to selectively process perceptual information.

In spatially incongruent conditions, accuracy was reduced across all delay lengths indicating the displaced hand receiving synchronous visuo-tactile input was embodied to some extent, in all groups. However, in contrast to the CA group, pointing accuracy was only significantly worse for the medium (180 ms) and long (300 ms) conditions for the ASD and MA group but not the shortest (60 ms) condition. Specifically, this suggests the MA and ASD groups do not seem to reliably detect and embody the synchronous hand when the delay applied to the asynchronous hand is only 60 ms. These results mirror the findings of Greenfield et al. (2015) suggesting that visuo-tactile processing in ASD is extended, but crucially the findings demonstrate perception of visuo-tactile synchrony impacts upon body schema, not just body representation. In addition, it further adds weight to the argument that the temporal binding window becomes more sensitive and specific with age (Hillock-Dunn and Wallace, 2012) as the younger MA group also showed reduced embodiment at shorter delays unlike older typically developing children (i.e. CA group).

Consistent with these findings, when congruency scores were compared across groups (i.e. spatially congruent minus incongruent condition, at each delay length) the ASD and MA groups had significantly lower scores, indicating reduced embodiment. This indicates that, the CA group embodied the synchronous hand more consistently than the other groups, which was likely driven by their reduced accuracy in pointing in the spatially incongruent condition. Additionally, a main effect of delay indicated that detection of the synchronous hand was most difficult in the shortest delay condition (60 ms) for all groups compared to the medium and longer delay conditions. This finding supports the premise that the extent to which we embody a hand, relies on our ability to distinguish synchronous from asynchronous visuo-tactile inputs.

Overall these results provide good evidence to support the role of temporal binding in the development of sensory integration processes in both typical and ASD populations. Importantly, the finding that identification of the synchronous hand as one’s own, can directly impact upon body schema (an unconscious representation of the body that is used for action and interaction with the environment) rather than just body image (a top-down, perceptual representation of the body). This finding is important in light of research suggesting these two processes may be distinct from one another (Haggard and Wolpert, 2005, Kammers et al., 2010). In addition, this suggests a link between sensory processing and action, which could impact upon the development of social processes. For example, infants learn that when they touch an object they can feel it (tactile information) at the same time as they see their hand touching it (visual information). Through this experience, they learn about the relationship between perception and action, which allows them to interpret and interact with their environment (Von Hofsten, 2004, Von Hofsten, 2007) and determine self versus other generated actions (Milward and Sebanz, 2016). If children with ASD have reduced sensitivity to the temporal constraints of sensory binding then this may inhibit or delay this experience-dependent learning, impacting upon the development of social processes such as empathy. Some evidence for a link between sensory integration and social processes was found in the current study through a significant positive correlation between Social Aptitude Scores and congruency effect (i.e. an indicator of embodiment). However, this correlation was small, and there was a lack of a significant relationship between congruency scores and another parental report measure of social functioning (i.e. Social Communication Questionnaire) therefore the finding must be interpreted with caution. It is possible that the measures of social ability in the current study were too general and may be less reliable as they both involved parental reports.

A stronger association between sensory and social symptoms may have been found using a more specific measure of social functioning which has a clear link with the sensory modalities being explored. Support for this argument comes from a study by Cascio et al. (2012) who demonstrated a relationship between susceptibility to the rubber hand illusion, which is induced through detection of visual-tactile synchrony, and a measure of empathy. Unfortunately, the method employed in this study was not able to present a number of differ visuo-tactile delays across multiple trials to determine temporal sensitivity. It will be valuable for future research to develop the current MIRAGE task further, and present it alongside a range of behavioural tasks designed to measure body representation and social functioning to better understand which areas it impacts upon.

A further question raised by the current findings is how extended visuo-tactile binding in ASD relates to sensory integration difficulties involving other modalities. Specifically, this work extends on research showing atypical temporal binding on visual-auditory processing in ASD (e.g. Foss-Feig et al., 2010, Kwakye et al., 2011, Stevenson et al., 2014, Woynaroski et al., 2013). Evidence in this area has not only shown extended temporal binding between auditory-visual information in autism, but also found it related to performance on a speech perception task (Stevenson et al., 2014). An important question that needs addressed is whether there is a general difficulty with temporal binding of sensory inputs that impacts upon a range of cross-modality pairings (e.g. visuo-tactile, visuo-auditory) or whether these may be selectively or differentially affected.

In addition, one needs to clarify how sensory integration difficulties can account for the hyper and hypo sensory symptoms reported through a number of clinical accounts in those with ASD. An inability to bind synchronously occurring inputs together could result in an individual processing each input as a separate event. Therefore, this could make ‘noisy’ environments, (i.e. those with a high degree of sensory information), such as a classroom, overwhelming and may lead to the avoidance of social situations. To reduce feelings of sensory overload, individuals with ASD may then chose to focus on information from one sensory modality at the expense of other modalities, leading to hypersensitivities to that sense and hypo-sensitivities to other sensory inputs (Bahrick and Todd, 2012). However, in some circumstances where hypersensitivity to a single sensory input is observed (Cascio et al., 2008), an account of low-level temporal sensory integration difficulties may not be as evident. Thus, we may also need to consider the role of higher-level processes such as predictive encoding (Bays et al., 2006) or attentional and inhibitory control (Marco et al., 2011) to fully account for sensory symptoms in ASD.

While extended temporal binding may offer a plausible explanation that could potentially account for social and sensory symptoms in ASD, one challenge is explaining why younger typically developing children who also show a less precise temporal processing do not show social difficulties to the same extent as those with ASD. It likely that the protracted period of development of the temporal binding window in ASD has a knock on effect on other processes resulting in more significant social difficulties in this population. Further research is needed to explore the relationship between extended temporal binding and a range of socio-cognitive skills to clarify the role of sensory integration in social processing across the developmental span, ideally with a longitudinal approach. In addition, future research needs to explore whether the delayed development of the visuo-tactile temporal binding processes observed in children with ASD remains or normalises in adulthood. Specifically, it is not clear whether extended visuo-tactile temporal binding is only seen in children with ASD, or whether it is also present in adults with the disorder (Paton et al., 2012). As the research has shown the temporal binding window is pliable and can be narrowed with training (Stevenson et al., 2013) this offers a potential avenue for the development of clinical interventions to address symptoms in ASD.

## Conflict of Interest

None.
